# Neuroprotective effect of Ziziphi Spinosae Semen on rats with p-chlorophenylalanine-induced insomnia *via* activation of GABA_A_ receptor

**DOI:** 10.3389/fphar.2022.965308

**Published:** 2022-11-22

**Authors:** Fengqin Xiao, Shuai Shao, Hongyin Zhang, Guangfu Li, Songlan Piao, Daqing Zhao, Guangzhe Li, Mingming Yan

**Affiliations:** ^1^ Northeast Asia Research Institute, Changchun University of Traditional Chinese Medicine, Changchun, China; ^2^ Jilin Provincial Science and Technology Innovation Center of Health Food of Chinese Medicine, Changchun University of Chinese Medicine, Changchun, China

**Keywords:** Ziziphi Spinosae Semen, insomnia, para-chlorophenylalanine, GABA_A_Rα1, GABA_A_Rγ2

## Abstract

*Ziziphus jujuba* var. spinosa (Bunge) Hu ex H.F*.*Chow [Rhamnaceae; Ziziphi Spinosae Semen (ZSS)] has attracted extensive attention as the first choice of traditional Chinese medicine in the treatment of insomnia. However, recent studies on the sleep-improving mechanism of ZSS have mainly focused on the role of single components. Thus, to further reveal the potential mechanism of ZSS, an assessment of its multiple constituents is necessary. In this study, ZSS extract (ZSSE) was obtained from ZSS *via* detailed modern extraction, separation, and purification technologies. The chemical constituents of ZSSE were analyzed by high-performance liquid chromatography–mass spectrometry (HPLC–MS). For *in vivo* experiments, a rat model of insomnia induced by p-chlorophenylalanine (PCPA) was established to investigate the potential effect and corresponding mechanism of ZSSE on improving sleep. Hematoxylin–eosin staining (HE) results revealed that the drug group showed prominent advantages over the model group in improving sleep. Moreover, the brain levels of γ-aminobutyric acid (GABA), glutamic acid (Glu), 5-hydroxytryptamine (5-HT), and dopamine (DA) were monitored *via* enzyme-linked immunosorbent assay (ELISA) to further study the sleep-improving mechanism of ZSSE. We found that sleep was effectively improved *via* upregulation of GABA and 5-HT and downregulation of Glu and DA. In addition, molecular mechanisms of ZSSE in improving sleep were studied by immunohistochemical analysis. The results showed that sleep was improved by regulating the expression levels of GABA receptor subunit alpha-1 (GABA_A_Rα1) and GABA acid receptor subunit gamma-2 (GABA_A_Rγ2) receptors in the hypothalamus and hippocampus tissue sections. Therefore, this work not only identified the active ingredients of ZSSE but also revealed the potential pharmacological mechanism of ZSSE for improving sleep, which may greatly stimulate the prospective development and application of ZSSE.

## Introduction

Recently, sleep disorders have become a growing health problem. Specifically, the global rate of sleep disorders has reached 27% according to World Health Organization (WHO) statistics. As a type of sleep disorder, insomnia has become one of the important factors affecting people’s quality of life. Insomnia may even lead to traffic accidents and other accidents and endanger personal and public safety, thus posing a serious burden to individuals and society. Therefore, the development of highly efficient drugs in treating insomnia to promote people’s physical and mental health is of critical importance. Clinically, benzodiazepines are candidate drugs for treating insomnia (McKillop et al., 2021). Generally, the total sleep time can be effectively increased by benzodiazepines by shortening the sleep latency period and reducing the number of awakenings during nighttime sleep (Lahteenmaki et al., 2019). However, the long-term use of benzodiazepines can lead to drowsiness (Qeadan et al., 2022), memory impairment (Kleinman and Weiss 2022), ataxia, drug addiction, and other adverse reactions (Murphy et al., 2016). Chinese medicine, such as Suanzaoren decoction, has shown unique advantages in the treatment of insomnia (Dong et al., 2021). Therefore, the development of natural and nontoxic Chinese herbal medicine to improve sleep function is highly desirable.

As the preferred medicine in treating insomnia in traditional Chinese medicine, ZSS has over 200 years of history of clinical application. ZSS contains saponins, flavonoids, fatty acids, alkaloids, proteins, and other chemical constituents (He et al., 2020). Benefits from the diversity of chemical constituents of ZSS, diversity of biological activity, and pharmacological action have been presented. Modern pharmacological studies have confirmed that ZSS exhibits a series of pharmacological effects, such as neuroprotective effects (Liu et al., 2014), antianxiety effects (Li et al., 2019), and sleep-improving effects (Fang et al., 2010). A previous report verified that ZSS saponins could enhance the hypnotic effect of pentobarbital sodium (Cao et al., 2010). Jujubosides A and B can improve the sleep of *Drosophila melanogaster* because jujuboside A mainly affects the autonomous activities of *Drosophila* at night, and jujuboside B mainly improves the daytime sleep of *Drosophila* (Yang Bo et al., 2013). Spinosin, a flavonoid in ZSS, assists pentobarbital to induce sleep time in mice and shortens sleep latency (Wang et al., 2010). ZSS oil can significantly reduce the number of autonomous activities of mice to prolong sleep time and shorten the sleep latency of mice, so as to further improve sleep quality (Jia et al., 2018). Total alkaloid gavage of ZSS can significantly prolong the sleep time of mice by reducing their spontaneous activity (Fu jingwei and chen, 2005). In summary, all the aforementioned constituents of ZSS have shown a good pharmacological effect on improving sleep. However, the active ingredients of ZSS and the corresponding sleep-improving mechanism are still unclear, which restricts the rapid development of ZSS medicine.

GABAergic neurons are widely distributed in the central nervous system and form neural networks in the brain. GABAergic neurons communicate with a variety of neurons through different receptor subtypes to jointly regulate the sleep–wake process (Li et al., 2022). GABA receptors are mainly divided into ionic-channel receptors [GABA receptor A (GABA_A_) and GABA receptor C (GABA_C_)] and metabolic receptors [GABA receptor B (GABA_B_)] (Wisden et al., 2019). Many anti-insomnia drugs currently used in clinical practice have been designed and developed as inhibitors of these GABA receptor targets (Belelli and Lambert 2005). Among them, the GABA_A_ receptor is the most abundant GABA receptor in the human brain; it is widely distributed in the hippocampus, hypothalamus, and cerebral cortex (Michels and Moss 2007). It is the most important of the three subtypes because it acts as the target of many major clinical anti-insomnia drugs, such as diazepam, barbiturates, clonazepam, and midazolam (Brickley and Mody 2012). The clinical efficacy of these drugs correlates well with their affinity for the GABA_A_ receptor (Ghit et al., 2021). Therefore, the development of anti-insomnia drugs targeting the GABA_A_ receptor has become a research hot spot.

Although the efficacy of ZSS has been widely recognized by doctors and patients, the underlying mechanism of improving sleep is still unclear. In this study, the ZSS extract (ZSSE), which was obtained from ZSS using the pharmacodynamic tracking method, was taken as the research object. Specifically, to clarify the pharmacodynamics material basis of ZSSE and to understand the underlying mechanisms of improving sleep, high-performance liquid chromatography (HPLC) and HPLC–mass spectrometry (MS) were performed to systematically analyze the compound composition of ZSSE. On this basis, according to the effective components and action targets obtained by molecular docking, we established a rat model of insomnia induced by PCPA to evaluate the sleep-improving effects of ZSSE and investigate the GABA anti-insomnia pathway for exploring the molecular mechanism. This study may provide new insights into the protective effects and molecular mechanism of ZSSE against insomnia.

## Materials and methods

### Animals

The effect of ZSS on sleep improvement was studied in specific pathogen-free (SPF) Kunming mice (body weight, 18–22 g). Six-week-old male Sprague Dawley (SD) rats (body weight, 180–220 g) were used to explore the mechanism of sedative–hypnotic effects of ZSSE on the PCPA-induced insomnia model. All animals were obtained from Liaoning Chang Sheng Biotechnology Co. Ltd. (License No. SCKK (Liao) 2020-0001). All experimental procedures performed in this work conformed to the ethical standards of the Medical Ethics Committee of Changchun University of Chinese Medicine, and every effort was made to reduce the number of animals used and any pain and discomfort they experienced.

### Cell culture

The mouse hippocampal neuron cell line, HT22 (American Type Culture Collection, Manassas, VA, United States), was grown in a DMEM supplemented with 100 U/mL penicillin, 100 mg/ml streptomycin, and 10% calf serum at 37°C in a humidified incubator with an atmosphere of 95% air and 5% CO_2_. All the reagents were dissolved in the cell culture medium.

### Reagents and instruments

Ziziphi Spinosae Semen (ZSS) was purchased from the Pharmacy Department of the Affiliated Hospital of Changchun University of Traditional Chinese Medicine in Changchun, Jilin Province. The purchased wild jujube seed was identified as the seed of *Zizyphus jujuba* Mill. var. spinosa (Bunge) Hu ex H.F.Chow by the School of Medicine of Changchun University of Traditional Chinese Medicine. The identified specimen was deposited in the College of Pharmacy, Changchun University of Traditional Chinese Medicine, Changchun, Jilin Province, China, with the specimen number C2020602. Sodium pentobarbital (Lot: F0749) was purchased from Shandong West Chemical Technology Co. Ltd. Diazepam (national drug standard: H37023039) was purchased from Shandong Xinyi Pharmaceutical Co., Ltd. PCPA was purchased from Alfa Aesar Company, while 5-HT, GABA, DA, and Glu ELISA kits; PV-6000 immunohistochemical kits; and GABA_A_Rα1 and GABA_A_Rγ2 antibodies were purchased from Beijing Solebao Technology Co., Ltd.

The instruments used in this experiment included KD-MBII biological tissue–embedding machine and Leica RM2245 paraffin slicer (Hubei Kanglong Electronic Technology Co., Ltd.), 10AO automatic photography device, optical microscope (Olympus, Japan), JY10001 electronic scale (Shanghai Precision Scientific Instrument Co., Ltd., China), Agilent-1260 HPLC (Agilent Technologies Inc.), UltiMate 3000RS-chromatograph, and Q Exactive high-resolution mass spectrometer (Semel Fisher Technology (China) Co., Ltd.).

### Screening of ZSSE for improving sleep

**FIGURE 1:**
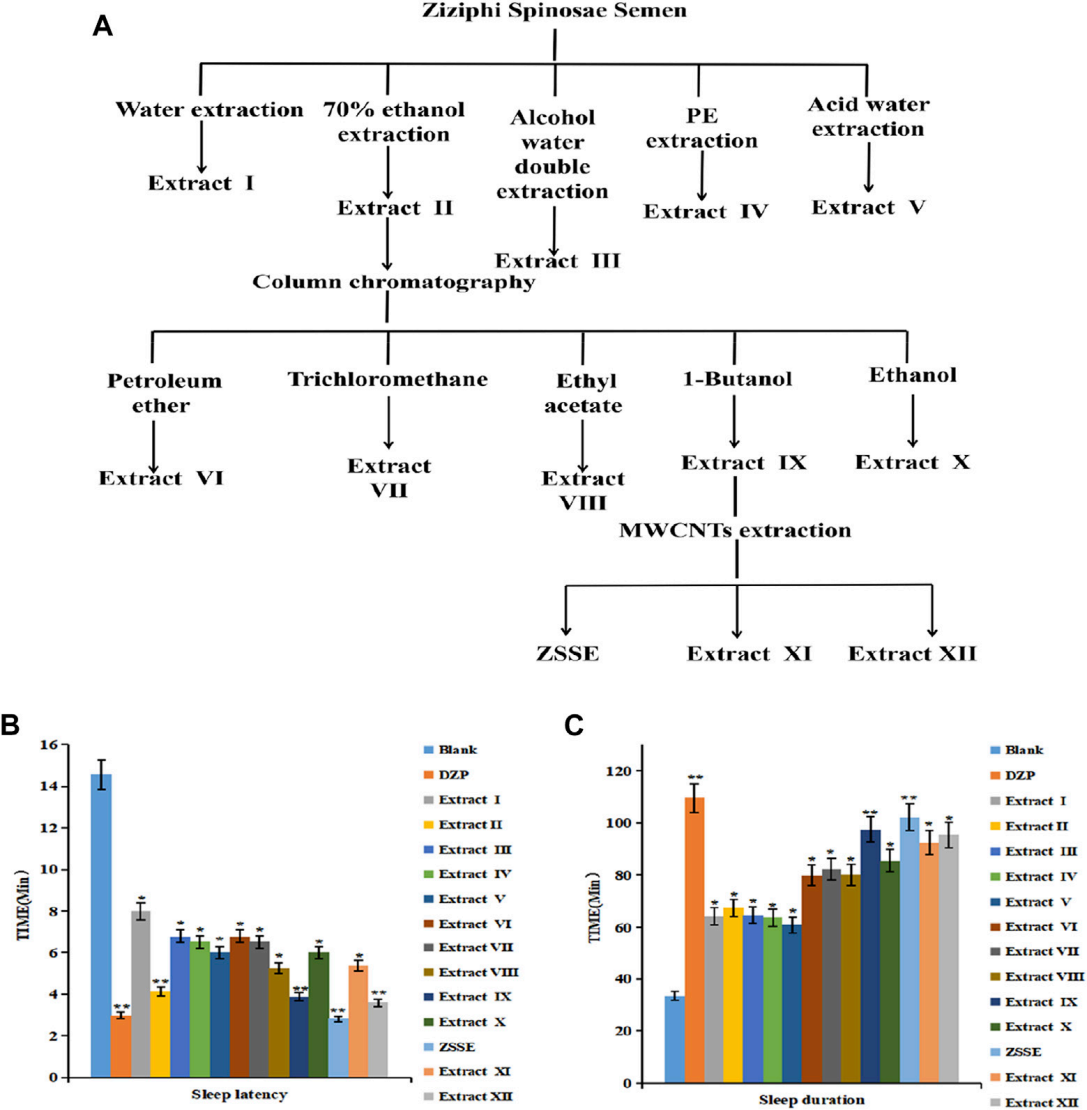
ZSS improves the screening of sleep extracts. ZSS improves sleep activity screening. Flow chart of the ZSS sleep improvement extract **(A)**; effects of ZSS extracts I–XII on sleep latency in mice **(B)**; effects of ZSS extracts I–XII on sleep duration in mice **(C)**.

The extraction method of ZSS is shown in Figure 1A. The effects of ZSSE on sleep latency and sleep duration of the mice were screened by a pentobarbital sodium threshold experiment, where the disappearance of righting reflex was an indicator of falling asleep. The contents of total saponins, polysaccharides, flavonoids, and fatty acids were determined by ultraviolet spectrophotometry (SHIMADZU UV1700, Kyoto, Japan).

### Preparation of ZSSE

After grinding, ZSS was degreased with petroleum ether to obtain powder. After conducting reflux extraction with 10 times 70% ethanol three times, 1 h each time, we collected the combined filtrate and dried it. We added n-butanol to the dried alcohol extract 10 times; conducted reflux extraction three times, 1 h each time; and filtered, collected the filtrate, recovered the solvent under reduced pressure, and then dried it. The dried substance was taken and 70% ethanol was added, and it was fully heated for ultrasonic dissolution to prepare the liquid medicine. A multi-walled carbon nanotube (MWCNT) was used to vortex it for 15 s and mix it well; a strong magnet was added to the outer wall of the test tube, and it was left to stand for 30 s; then, the supernatant was poured into a test tube. n-Butanol was added to the aforementioned test tube containing a MWCNT for elution (vortex for 15 s, ultrasonic for 15 min), the eluent was collected, the solvent was recovered under reduced pressure, and then it was dried. The dried product is ZSSE.

### Identification of chemical components in ZSSE by LC-MS

On the Ultimate 3000RS chromatographic system, the chemical composition of the ZSSE sample solution was analyzed and identified by high-resolution LC-MS. MS conditions were as follows: ion source, electrospray ionization source; scanning mode, positive and negative ion scanning switch; detection method, full mass/dd-MS2; resolution, 70,000 (Fullmass) and 17,500 (dd-MS2); scanning range,: 150.0 kV (positive); capillary temperature, 300°C; collision gas, high-purity argon (purity ≥99.999); and sheath, nitrogen (purity ≥99.999, 40 arb). Chromatographic conditions were as follows: RP-C18 column (150 mm × 2.1 mm, 5 μm Welch); flow rate of 0.30 ml/min; A phase, 0.1% formic acid aqueous solution; B phase, 0.1% formic acid acetonitrile; needle lotion, methanol; column temperature, 35°C; injection volume, 5 μl; and solvent gradient as shown in Table 1.

**TABLE 1:** Gradient elution procedures.

Time (min)	A: 0.1% formic acid aqueous solution (%)	B: 0.1% formic acid acetonitrile (%)
0	98	2
1	98	2
5	80	20
10	50	50
15	20	80
20	5	95
25	5	95
26	98	2
30	98	2

### Determination of ZSSE chemical composition by HPLC

Fifteen batches of ZSSE samples were analyzed by an Agilent-C18 column (250 mm × 4.6 mm, 5 μm) on the Agilent-1260 HPLC system. The mobile phase system consisted of mobile phase A (acetonitrile) and mobile phase B (0.1% phosphate water). The solvent gradient is shown in Table 2. The chromatographic conditions were as follows: detection wavelength, 250 nm; column temperature, 25°C; flow rate, 1 ml/min; and injection volume, 20 μl.

**TABLE 2:** Gradient elution schedule.

Time (min)	A: Acetonitrile (%)	B: 0.1% phosphate water (%)
0	10	90
30	16	84
50	25	75
65	40	60
75	40	60

### Docking procedure

Molecular docking is a method of placing the ligand in the binding area of the receptor through computer simulation and calculating its physical and chemical parameters to predict the binding affinity and conformation of the ligand and receptor (Morris and Corte 2021). The GABA_A_ receptor, the target of diazepam and barbiturates widely used in clinical practice (Sigel 2005), was selected for molecular docking with ZSSE’s active ingredients. Through the analysis of the docking score, the results were obtained and evaluated. Corresponding gene target proteins were selected through the RSCB PDB database (https://www.rcsb.org/), and 3D structure PDB format files were downloaded. We downloaded the MOL_2_ file of the corresponding active ingredient in the TCMSP database. The target protein and small-molecule ligand were processed by AutoDock software. AutoDock Vina was used to calculate the binding energy. Data visualization was performed using Pymol software.

### Effect of ZSSE on the activity of HT22 cells

The effect of ZSSE on the activity of HT22 cells injured by Glu was determined by the Cell Counting Kit-8 (CCK-8) method. HT22 cells were spread in 96-well plates at the concentration of 5 × 10^3^ cells/well. After 24 h of culture, the cells were divided into the following seven groups: blank group; Glu group; and 25, 50, 100, 200, and 400 μg/ml ZSSE administration groups. The administration group was given 25, 50, 100, 200, or 400 μg/ml ZSSE pretreatment for 3 h. L-Glu was added to the cells of the model group and the administration group to achieve the final concentration of 20 mM, and the cells were incubated for 24 h. Then, 10 µl CCK-8 was added to each well and cultured under the same conditions for 2 h. Optical density (OD) was measured at 490 nm using a Bio-Rad 550 microplate reader. The reference wavelength was 620 nm. The experiment was repeated three times. The effect of ZSSE on the activity of HT22 cells induced by Glu was calculated as follows:

Cell viability (%) = D_1_/D_0_ × 100%, where D_0_ is the OD value of the control wells, and D_1_ is the OD value of the sample wells.

### Preparation of the PCPA-induced insomnia rat model

After the rats were adaptively reared for 7 days, they were injected intraperitoneally with 350 mg/kg PCPA once a day for three consecutive days. The animals showed a loss of circadian rhythm and were active during the day, suggesting that the insomnia model was successfully established. Sixty rats were successfully modeled and divided into the model group (MOD); diazepam group (DZP, 1.5 mg/kg); jujuboside B group (JUB, 20 mg/kg); and ZSSE 13.50 mg/kg, 9.01 mg/kg, and 4.50 mg/kg groups. Another 10 unmodeled rats were selected as the blank control group (Blank). All of the groups received intragastric administration once a day for 7 days (Figure 6A).

### Histopathological analysis

Four hours after the last administration, the rats were given deep anesthesia. After anesthesia, normal saline was perfused through the heart until the effluent was colorless, and then 4% paraformaldehyde was perfused. The brain was quickly taken out and placed on an ice-cold Petri dish. The blood was washed with ice saline and fixed in a 10% neutral formaldehyde solution for 24 h. Then, the brain tissue was dissected and immersed in different gradients of ethanol and xylene for dehydration. After dehydration, the tissue was embedded in paraffin and sectioned in 4-μm-thick coronal slices. Histomorphological changes of the hypothalamus and hippocampus of rats with insomnia were observed by hematoxylin and eosin (HE) staining under an optical microscope (×100).

### Biochemical analysis

The experimental rats were deeply anesthetized 4 h after the last administration, and the hypothalamus and the hippocampus were quickly removed. Nine times normal saline was added to the hypothalamus and hippocampus tissue, which was then prepared into a homogenate using a high-speed disperser. The homogenate was centrifuged at 3,500 r/min for 10 min to collect the supernatant. The contents of 5-HT, Glu, DA, and GABA in the hypothalamus and hippocampus tissue homogenate were determined by ELISA. The possible mechanisms of the sedative–hypnotic effect of ZSSE were investigated by comparing 5-HT, DA, Glu, and GABA contents of the hypothalamus and hippocampus tissues of rats between the groups.

### Immunohistochemistry analysis

The sections were deparaffinized and rehydrated in xylene and different grades of alcohol. Antigen retrieval was performed by heating for 60 min. The sections were incubated with 3% H_2_O_2_ deionized water for 10 min to block the activity of endogenous peroxidase. After being washed with phosphate buffered saline (PBS) three times, we used an antibody diluent blocking buffer to block the non-specific binding site for 1 h at room temperature. The primary antibody (1:200) was added to the slides and incubated at 37°C for 2 h. After washing with PBS, we added the secondary antibody to the slices and incubated them at 37°C for 30 min. Then, the slices were washed three times with PBS, for 5 min each time. The slides were then exposed to the 3,4,3′,4′-tetraaminodiphenyl (DAB) substrate following the kit instructions. The stained slides were dehydrated, transparent, and sealed. The mean optical density (MOD) of GABA_A_Rα1- and GABA_A_Rγ2-positive cells in the cerebral cortex was determined by light microscopy.

### Statistical analysis

All experiments were conducted at least three times. The data were reported as mean value ± standard deviation. Statistical analyses were conducted using SPSS 21.0 and Origin 9.0. For multiple comparisons, data were subjected to one-way ANOVA (Turkey’s *post-hoc* test) and paired *t*-test to determine the level of statistical significance. *p* < 0.05 was considered statistically significant.

## Results

### ZSS improves sleep

As shown in Figure 1A, 13 ZSS extracts were obtained by solvent extraction with different solvents. The yield of ZSSE was 1%. The pentobarbital sodium threshold test was used to screen the sleep-improving activity of these 13 extracts. The results are shown in Figures 1B,C. Specifically, compared with other extracts, ZSSE significantly prolonged the sleep duration of mice and shortened the sleep latency of mice, indicating that ZSSE had a good effect on improving sleep (Table 3). In addition, the contents of total saponins, total flavonoids, total alkaloids, and total fatty acids in ZSSE were determined by UV spectrophotometry. The results showed that ZSSE contained a large amount of saponins and fatty acids (Table 4).

**TABLE 3:** Effects of ZSS extract on sleep latency and sleep duration in mice (n = 10, mean ± S.D.).

Group	Doses	Sleep latency	Sleep duration
Blank	___	14.56 ± 5.76	33.36 ± 11.20
DZP	1.50 mg/kg	2.98 ± 1.43^a^	109.58 ± 16.76^a^
Extract I	1.3 g/kg	8.00 ± 5.07^b^	64.18 ± 11.95^b^
Extract II	1.3 g/kg	4.12 ± 0.64^a^	67.42 ± 13.18^b^
Extract III	1.3 g/kg	6.79 ± 6.11^b^	64.71 ± 16.69^b^
Extract Ⅳ	1.3 g/kg	6.50 ± 4.37^b^	63.62 ± 15.20^b^
Extract Ⅴ	1.3 g/kg	5.98 ± 3.5^6^ ^b^	60.82 ± 12.49^b^
Extract Ⅵ	1.3 g/kg	6.79 ± 6.11^b^	79.87 ± 20.01^b^
Extract Ⅶ	1.3 g/kg	6.50 ± 4.37^b^	82.18 ± 17.64^b^
Extract Ⅷ	1.3 g/kg	5.24 ± 1.75^b^	80.15 ± 19.70^b^
Extract Ⅸ	1.3 g/kg	3.87 ± 3.47^a^	97.39 ± 12.89^a^
Extract Ⅹ	1.3 g/kg	5.98 ± 3.56^b^	85.49 ± 12.53^b^
ZSSE	1.3 g/kg	2.80 ± 1.92^a^	95.34 ± 9.63^b^
Extract XI	1.3 g/kg	5.36 ± 2.77^b^	92.41 ± 11.82^b^
Extract XII	1.3 g/kg	3.58 ± 2.03^a^	112.22 ± 10.88^a^

Note: Compared with the Blank group.

^a^ *p*<0.01.

^b^ *p*<0.05.

**TABLE 4:** ZSSE active ingredient content table.

Plant	Total saponin content (mg GRE g^−1^)	Total flavonoid content (mg RUE g^−1^)	Total alkaloid content (mg ACE g^−1^)	Total fatty acid content (mg FTE g^−1^)
ZSSE	6.851 ± 0.062	2.114 ± 0.041	0.985 ± 0.005	5.833 ± 0.095

Note: Data are represented as means ± SD (n = 3). GRE: ginsenoside re equivalents; RUE: rutin equivalents; ACE: aconitine equivalents; FTE: fatty acid triglycerides equivalents.

### HPLC-MS analysis

**FIGURE 2:**
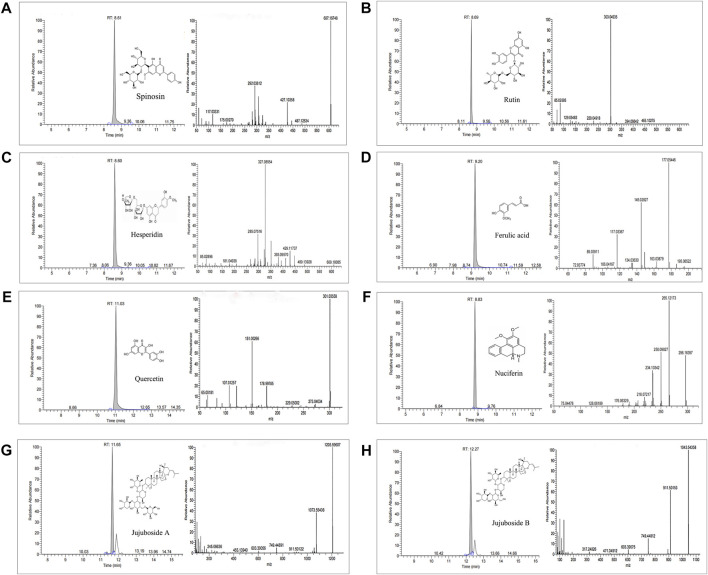
Spectrogram of the main compounds in ZSSE identified by UPLC-Q-Orbitrap-MS mass spectrometry. Spinosin in ZSSE**(A)**; rutin in ZSSE**(B)**; hesperidin in ZSSE **(C)**; ferulic acid in ZSSE **(D)**; quercetin in ZSSE **(E)**; nuciferin in ZSSE**(F)**; jujuboside A in ZSSE**(G)**; jujuboside B in ZSSE**(H)**.

The chemical constituents of ZSSE were analyzed by HPLC-MS. The molecular ion peaks (M-H^−^) and (M + H^+^) of each chromatographic peak in the positive and negative ion mode were detected by UPLC-Q-Orbitrap-MS, and the possible molecular weight was determined by the molecular ion peak, fragment ions, and their chromatographic peak retention time. As shown in Table 5, a total of 34 compounds in ZSSE were preliminarily identified, including spinosin, rutin, ferulic acid, quercetin, jujuboside A, and jujuboside B. A secondary mass spectrogram of the main compounds in ZSSE is shown in Figure 2.

**TABLE 5:** List of ZSSE compounds identified by UPLC-Q-Orbitrap-MS mass spectrometry.

NO.	Component name	Formula	Molecular weight	tR/min	Area	[M + H]+1	[M-H]-1
1	α-D-mannose 1-phosphate	C_6_ H_13_ O_9_ P	260.0293	1.79	2141838.22	--	259.02
2	Norharman	C11 H8 N2	168.07	6.44	793182.18	169.08	--
3	Spinosin	C28H32 O15	608.54	8.61	17651166.00	--	607.30
4	Rutin	C27H30 O16	610.15	8.69	84420324.00	--	609.14
5	Hesperidin	C28H34 O15	610.56	8.60	334840273.00	--	609.18
6	Nuciferin	C_19_H_21_NO_2_	295.37	8.83	71234552.03	296.1645	--
7	Ferulic acid	C10H10 O4	194.19	9.20	138552103.00	--	193.05
8	Quercetin	C15H10 O7	302.04	11.03	43616832.00	303.03	--
9	Jujuboside A	C58H94 O26	1207.35	11.65	89441488.00	--	1205.21
10	Citroflex 2	C_12_ H_20_O_7_	276.12	11.78	31068100.02	277.13	--
11	Isorhamnetin	C_16_ H_12_O_7_	316.06	12.07	1654689.90	317.07	--
12	Jujuboside B	C52H84 O21	1045.21	12.27	130876714.00	--	1043.54
13	Glycitein	C16 H12O5	284.07	13.74	2513278.92	285.07	--
14	Bis(4-ethylbenzylidene) sorbitol	C_24_H_30_O_6_	414.20	14.76	5266759.95	415.21	--
15	(+/-)12 (13)-DiHOME	C_18_H_34_O_4_	296.2344	14.94	5208181.23	297.24	--
16	α-Linolenic acid	C18H30 O2	278.22	16.09	8284980.70	279.23	--
17	Palmitoleic acid	C16H30 O2	254.22	16.61	1307156.50	255.23	--
18	16-Hydroxyhexadecanoic acid	C16 H32 O3	272.23	16.70	2382085.21	--	271.23
19	Dibutyl phthalate	C16 H22O4	278.15	17.56	156100574.70	279.16	--
20	Palmitoyl ethanolamide	C_18_H_37_ NO_2_	299.28	18.61	22198505.87	300.29	--
21	Pinolenic acid	C18H30O2	278.22	18.91	22576128.92	279.23	--
22	1-Linoleoyl glycerol	C21H38 O4	336.27	19.03	22630965.85	337.27	--
23	Ursolic acid	C30 H48 O3	456.36	19.20	182129584.80	--	455.35
24	Ethyl palmitoleate	C18 H34 O2	282.26	19.48	2865584.15	--	283.26
25	Oleamide	C18H35NO	281.48	19.88	81821910.05	--	265.25
26	Monoolein	C_21_H_40_O_4_	356.2917	20.33	44907121.30	--	357.29
27	Ethyl myristate	C16H32 O2	256.24	21.08	5451411.39	--	255.23
28	Oleic acid	C_18_H_34_O_2_	282.26	21.35	203048809.08	--	281.25
29	Linolenic acid ethyl ester	C20H34 O2	306.26	22.15	1411190.83	307.26	--
30	Ricinoleic acid methyl ester	C_19_H_36_O_3_	294.26	22.53	2310061.40	295.26	--
31	Stearic acid	C18H36 O2	284.27	22.86	8075045.05	--	283.26
32	Erucamide	C22 H43NO	337.33	24.05	2135618.78	--	338.34
33	Ethyl oleate	C_20_H_38_O_2_	310.29	24.91	310.29	311.29	--
34	4-Dodecylbenzenesulfonic acid	C18 H30 O3 S	326.19	27.51	5494426.33	--	325.18

### HPLC analysis

**FIGURE 3:**
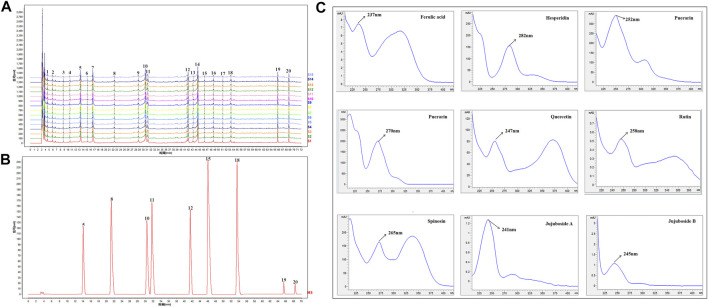
HPLC profile and quality control analysis of ZSSE using detection at 250 nm. The reproducible HPLC chromatograms of ZSSE from 15 batches **(A)**. HPLC chromatogram of ZSSE with nine peaks determined by comparing the retention time with the standards **(B)**. UV/DAD spectra of the standard mixture solution **(C)**. External standards, comprising puerarin (No. 5), ferulic acid (No. 8), spinosin (No. 10), rutin (No. 11), hesperidin (No. 12), nuciferin (No. 15), quercetin (No. 18), jujuboside A (No. 19), and jujuboside B (No. 20).

HPLC was carried out to investigate ZSSE. As shown in Figure 3A, a total of 15 typical ZSSE chromatograms with good quality control lots were observed. Furthermore, as shown in Figure 3B, nine peaks of ZSSE, namely, puerarin (No. 5), ferulic acid (No. 8), spinosin (No. 10), rutin (No. 11), hesperidin (No. 12), nuciferin (No. 15), quercetin (No. 18), jujuboside A (No. 19), and jujuboside B (No. 20), were identified in comparison with the corresponding reference standards. The relative contents of individual constituents in the nine peaks of ZSSE are shown in Table 6.

**TABLE 6:** Identification and determination of the compounds in the ZSSE by HPLC.

Number	Retention time of control substance (min)	Retention time of ZSSE sample (min)	Compound	Content (mg/g)
5	13.47 ± 0.04	13.34 ± 0.04	Puerarin	0.069 ± 0.003
8	22.26 ± 0.12	22.37 ± 0.06	Ferulic acid	0.026 ± 0.001
10	30.65 ± 0.09	30.64 ± 0.05	Spinosin	0.156 ± 0.005
11	21.21 ± 0.07	31.25 ± 0.06	Rutin	0.035 ± 0.008
12	41.52 ± 0.02	41.99 ± 0.08	Hesperidin	0.050 ± 0.002
15	48.92 ± 0.20	48.82 ± 0.41	Nuciferin	0.011 ± 0.005
18	53.51 ± 0.19	53.32 ± 0.05	Quercetin	0.024 ± 0.011
19	65.47 ± 0.09	65.70 ± 0.01	Jujuboside A	0.837 ± 0.013
20	68.38 ± 0.11	68.65 ± 0.01	Jujuboside B	0.654 ± 0.024

### Molecular docking

**FIGURE 4:**
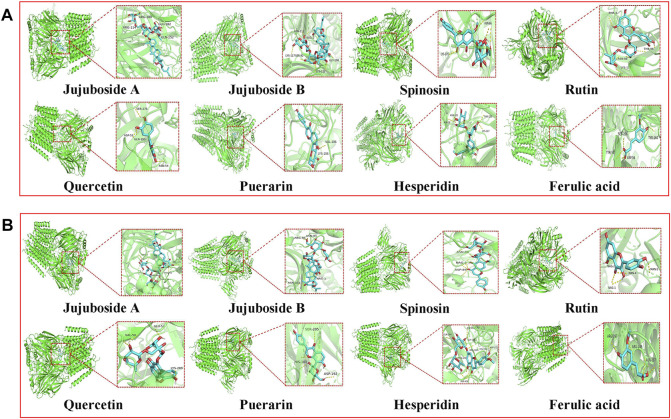
Three-dimensional docking analysis of the active components in ZSSE and two important targets for insomnia treatment. Active ingredient and target GABA_A_Rα1 molecular docking results **(A)**; active ingredient and target GABA_A_Rγ2 molecular docking results **(B)**.

The GABA_A_ receptor is the target of diazepam and barbiturate drugs widely used in clinical practice. Currently, among 16 types of GABA_A_ receptor subunits, α1 and γ2 GABA_A_ receptor subtypes are the most abundant ones in the brain, especially in the hippocampus and cerebral cortex, and they account for about 43% of all GABA_A_ receptors. Therefore, we chose GABA_A_Rα1 and GABA_A_Rγ2 receptors for molecular docking verification. The molecular docking results showed that eight of the aforementioned nine chemical components were related to GABA_A_Rα1 and GABA_A_Rγ2 receptors with a good binding ability (Figure 4 and Table 7). Jujuboside B exhibited the best combination ability with GABA_A_Rα1 (−10.5 kJ/mol), and the order from strong to weak was as follows: jujuboside B > jujuboside A > rutin > hesperidin > spinosin > puerarin > quercetin > ferulic acid. Jujuboside B exhibited the best combination ability with GABA_A_Rγ2 (−11.2 kJ/mol), and the order from strong to weak was as follows: jujuboside B > jujuboside A > puerarin > spinosin > hesperidin > rutin > quercetin > ferulic acid. All the results indicated that jujuboside B and jujuboside A from ZSSE exhibited the best binding ability with GABA_A_Rα1 and GABA_A_Rγ2 receptors, so they may play the main pharmacological role in improving sleep.

**TABLE 7:** Docking results of important components and target analysis.

Compound	Binding energy (kJ/mol)	Compound	Binding energy (kJ/mol)
	GABA_A_Rα1		GABA_A_Rγ2
Jujuboside B	−10.5	Jujuboside B	−11.2
Jujuboside A	−10.1	Jujuboside A	−9.8
Rutin	−9.7	Puerarin	−8.8
Hesperidin	−9.5	Spinosin	−8.4
Spinosin	−8.7	Hesperidin	−8.4
Puerarin	−7.8	Rutin	−8.3
Quercetin	−7.7	Quercetin	−7.6
Ferulic acid	−7.6	Ferulic acid	−6.4

### Effect of ZSSE on the survival rate of HT22 nerve cells induced by Glu

**FIGURE 5:**
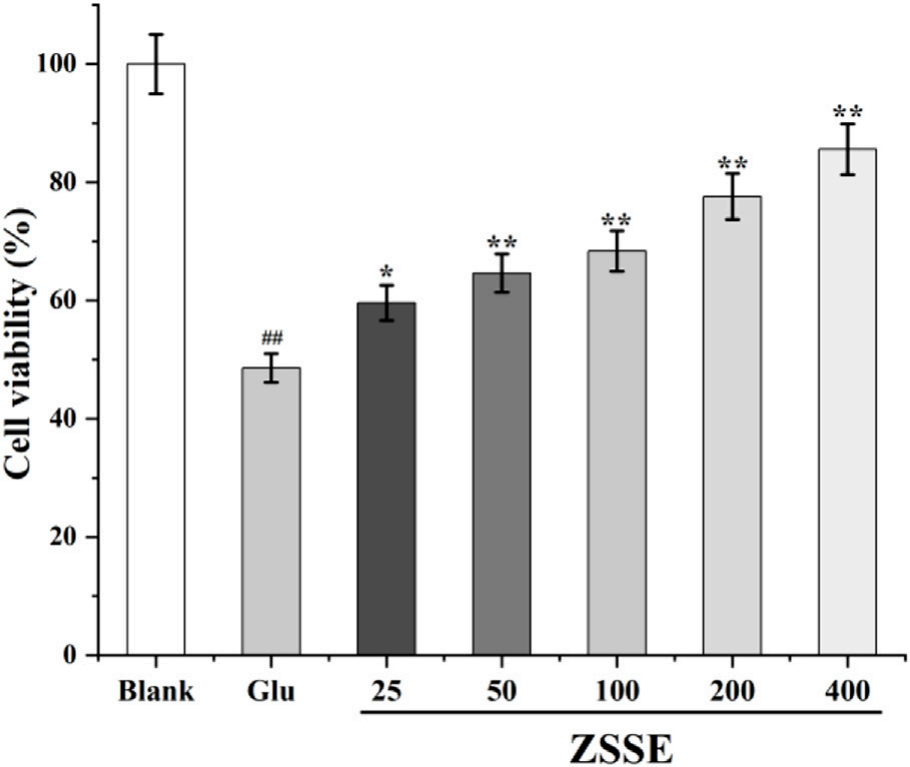
Protective effect of ZSSE on HT22 cells induced by Glu. Blank: normal control, normal medium culture; Glu: L-Glu was added to the cells to achieve the final concentration of 20 mM; ZSSE: the administration group was given 25, 50, 100, 200, or 400 μg/ml ZSSE pretreatment for 3 h. L-Glu was added to the cells of the model group and the administration group to achieve the final concentration of 20 mM.

As shown in Figure 5, 24 h after Glu modeling, the survival rate of cells in the Glu group decreased compared with that in the blank group. After ZSSE administration, the cell survival rate was significantly higher than that in the Glu group. The survival rate increased with the concentration of the drug. The results showed that ZSSE was able to reduce the damage to HT22 cells caused by Glu.

### Histopathological findings

**FIGURE 6:**
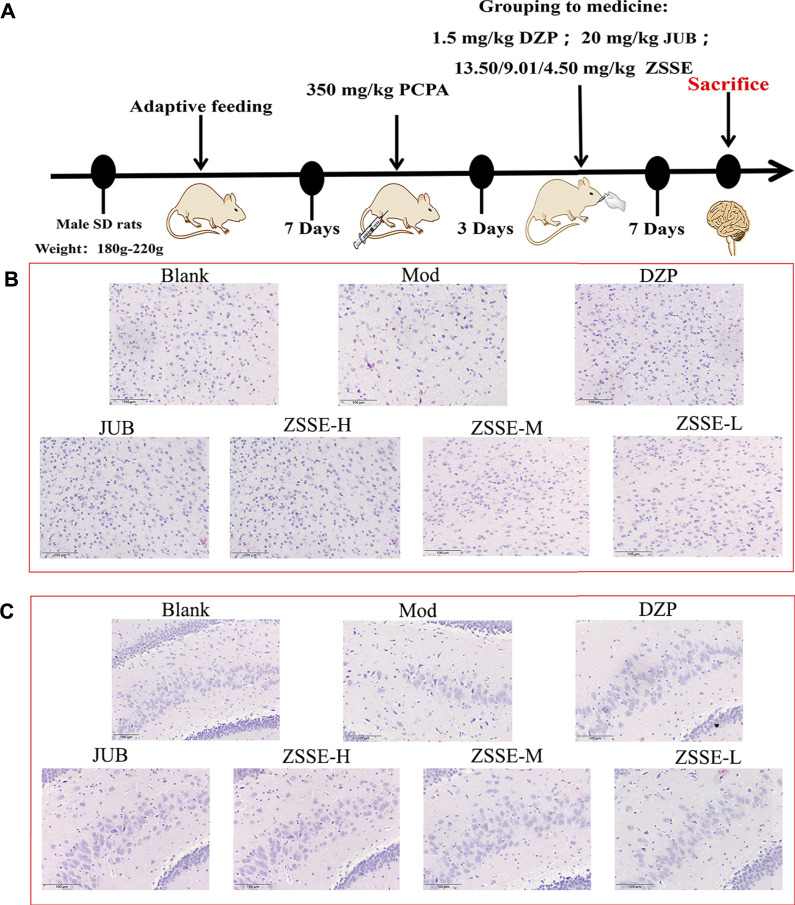
Pathological examination results of PCPA-induced insomnia rats by ZSSE (n = 5). Preparation of the PCPA-induced insomnia rat model **(A)**; histopathological findings of hypothalamic neuron degeneration and thalamic nucleus neuron atrophy **(B)**; histopathological findings of disordered and loosened hippocampal pyramidal cells with the unclear neuronal structure **(C)**. Blank: normal control, normal rats administered with 0.9% saline; Mod: PCPA-induced insomnia in rats, administered with 0.9% saline; DZP: PCPA-induced insomnia in rats, administered with 1.5 mg/kg/d diazepam; JUB: PCPA-induced insomnia in rats, administered with 20 mg/kg/d jujuboside B; ZSSE-H: PCPA-induced insomnia in rats, administered with 13.5 mg/kg/d ZSSE; ZSSE-M: PCPA-induced insomnia in rats, administered with 9.01 mg/kg/d ZSSE; ZSSE-L: PCPA-induced insomnia in rats, administered with 4.50 mg/kg/d ZSSE.

PCPA can selectively inhibit tryptophan hydroxylase in the brain and lead to insomnia by significantly reducing the 5-HT level in the brain. Within 26–30 h of modeling, the circadian rhythm of rats disappeared, which was accompanied by near-complete sleep deprivation (Yang et al., 2021; Shen et al., 2022). In this work, the rat insomnia model induced by PCPA was established to study the sedative and hypnotic mechanism of ZSSE. As shown in Figure 6, the HE staining results revealed that compared with the normal group, substantial neuronal degeneration was observed in the hypothalamus of the model group, and the neurons of the thalamic nuclei were atrophic. Furthermore, the hippocampal pyramidal cells appeared disordered and loose, with unclear neuronal structure. The aforementioned experimental results showed that the rat model of insomnia induced by PCPA was successfully established. After 1 week of administration intervention, compared with the model group, most of the nerve cells in the hypothalamus and hippocampus were recovered in the ZSSE administration groups, indicating that ZSSE was able to improve sleep by improving the damage to the rat hypothalamus and hippocampus caused by PCPA.

### Effects of ZSSE on GABA, Glu, 5-HT, and DA levels in the hypothalamus and hippocampus of rats

**FIGURE 7:**
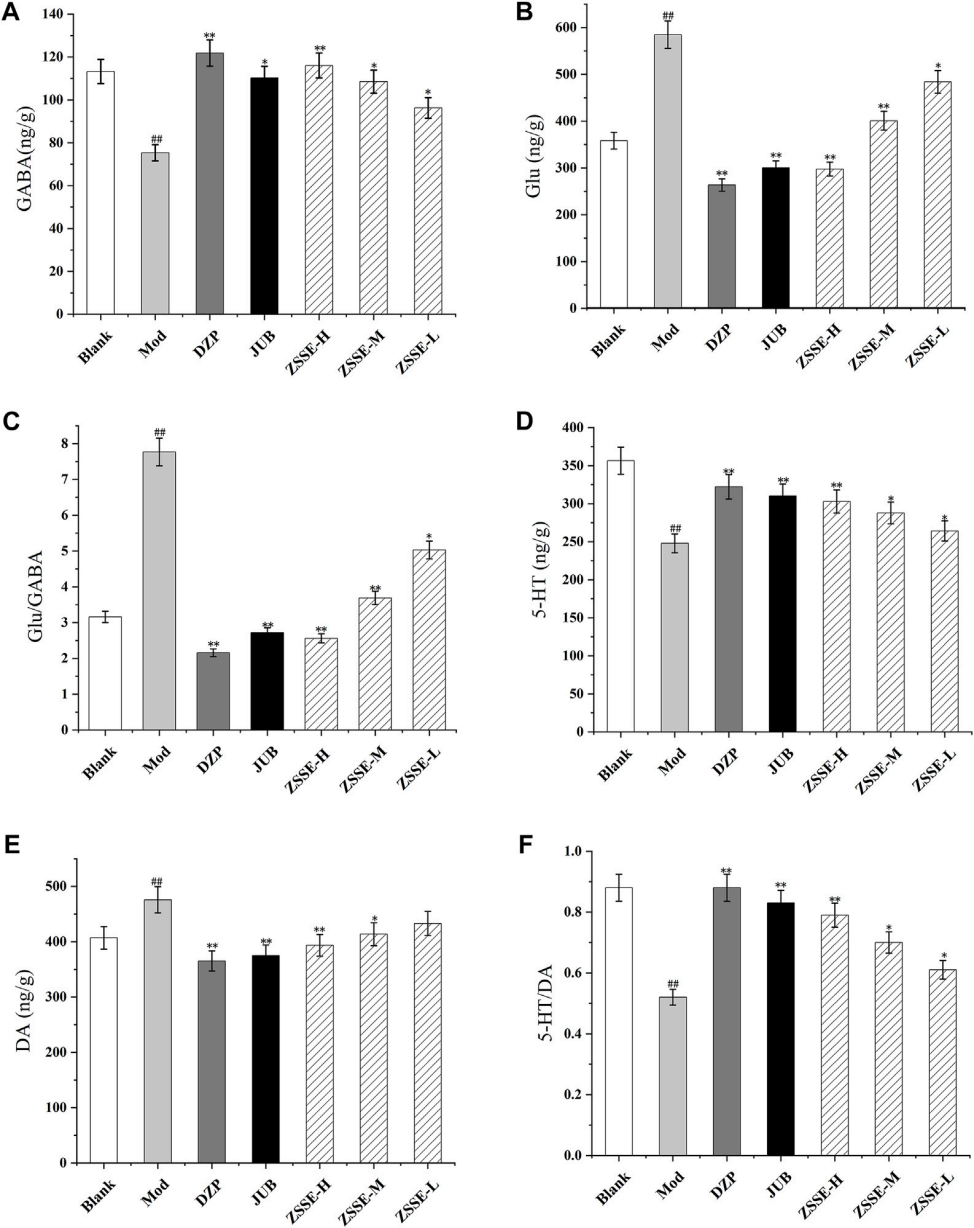
Effects of ZSSE on the amino acid and monoamine neurotransmitters in the hypothalamus of PCPA-induced insomnia rats. GABA **(A)**; Glu **(B)**; Glu/GABA **(C)**; 5-HT **(D)**; DA **(E)**; 5-HT/DA **(F)**; blank: normal control, normal rats administered with 0.9% saline; Mod: PCPA-induced insomnia in rats, administered with 0.9% saline; DZP: PCPA-induced insomnia in rats, administered with 1.5 mg/kg/d diazepam; JUB: PCPA-induced insomnia in rats, administered with 20 mg/kg/d jujuboside B; ZSSE-H: PCPA-induced insomnia in rats, administered with 13.5 mg/kg/d ZSSE; ZSSE-M: PCPA-induced insomnia in rats, administered with 9.01 mg/kg/d ZSSE; ZSSE-L: PCPA-induced insomnia in rats, administered with 4.50 mg/kg/d ZSSE. Data are presented as mean ± S.D. (n = 5). Compared with the Blank group ^*^ *p* < 0.05, ^**^ *p* < 0.01; compared with the model group ^#^ *p* < 0.05, ^#^ *p* < 0.01.

**FIGURE 8:**
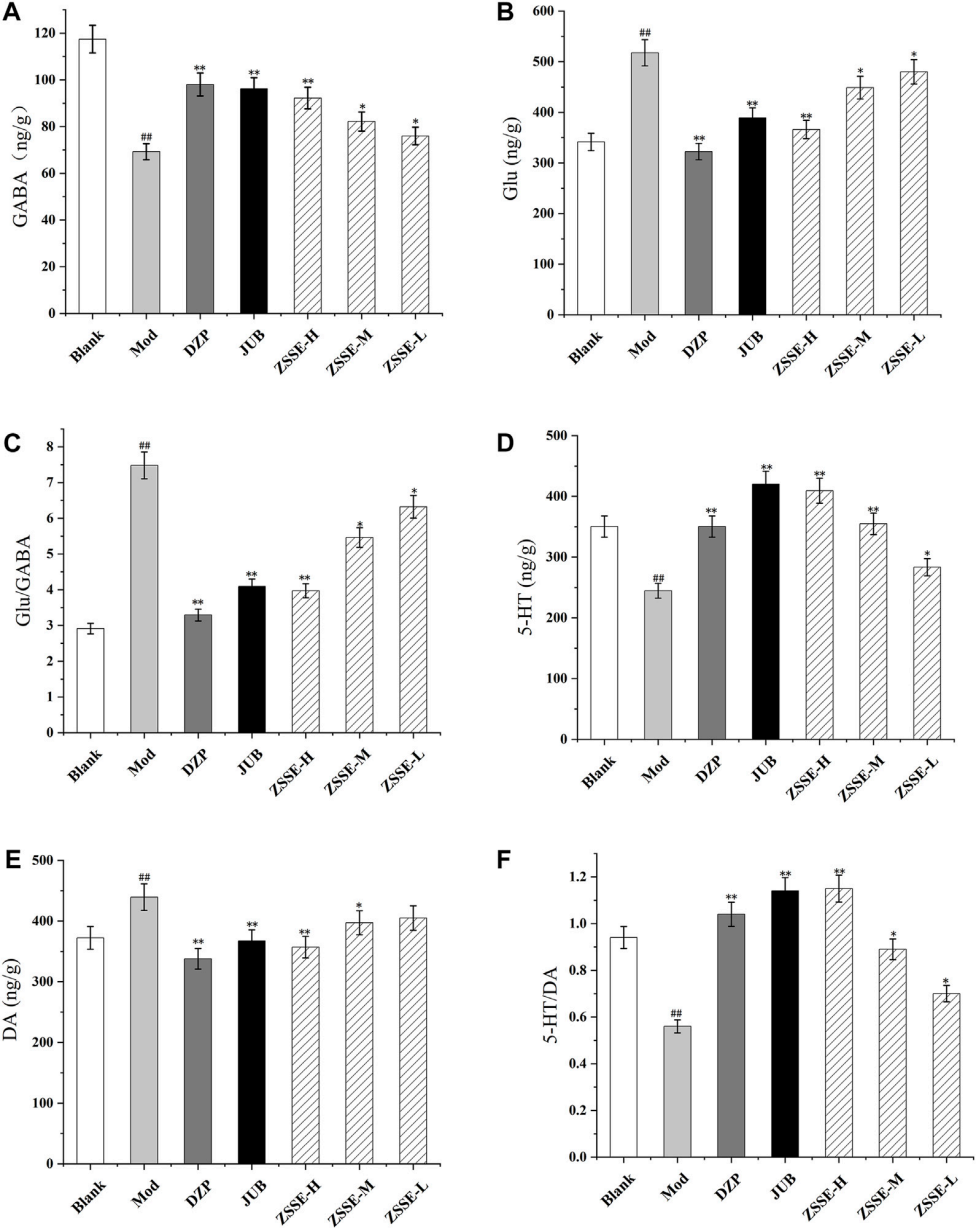
Effects of ZSSE on the amino acid and monoamine neurotransmitters in the hippocampus of PCPA-induced insomnia rats. GABA **(A)**; Glu **(B)**; Glu/GABA **(C)**; 5-HT **(D)**; DA **(E)**; 5-HT/DA **(F)**; Blank: normal control, normal rats administered with 0.9% saline; Mod: PCPA-induced insomnia in rats, administered with 0.9% saline; DZP: PCPA-induced insomnia in rats, administered with 1.5 mg/kg/d diazepam; JUB: PCPA-induced insomnia in rats, administered with 20 mg/kg/d jujuboside B; ZSSE-H: PCPA-induced insomnia in rats, administered with 13.5 mg/kg/d ZSSE; ZSSE-M: PCPA-induced insomnia in rats, administered with 9.01 mg/kg/d ZSSE; ZSSE-L: PCPA-induced insomnia in rats, administered with 4.50 mg/kg/d ZSSE. Data are presented as mean ± S.D. (n = 5). Compared with the Blank group ^*^ *p* < 0.05, ^**^ *p* < 0.01; compared with the model group ^#^ *p* < 0.05, ^#^ *p* < 0.01.

As shown in Figures 7 and 8, the contents of GABA and 5-HT in the hypothalamus and the hippocampus in the model group decreased significantly in comparison with the blank group, while the contents of Glu and DA increased significantly. Furthermore, as shown in Figures 7 and 8, compared with the blank group, the ratio of 5-HT/DA in the model group decreased significantly and the ratio of Glu/GABA increased significantly. All the results indicated that the model group was established successfully. Notably, compared with the model group, the concentrations of Glu and DA in the hypothalamus and the hippocampus were lower after administration of ZSSE, and the levels of GABA and 5-HT were higher. Compared with the model group, the ratio of 5-HT/DA in the hypothalamus and the hippocampus increased significantly, and the ratio of Glu/GABA decreased significantly.

### Effect of ZSSE on the expression of GABA_A_Rα1 and GABA_A_Rα1γ2 in the hypothalamus of rats

**FIGURE 9:**
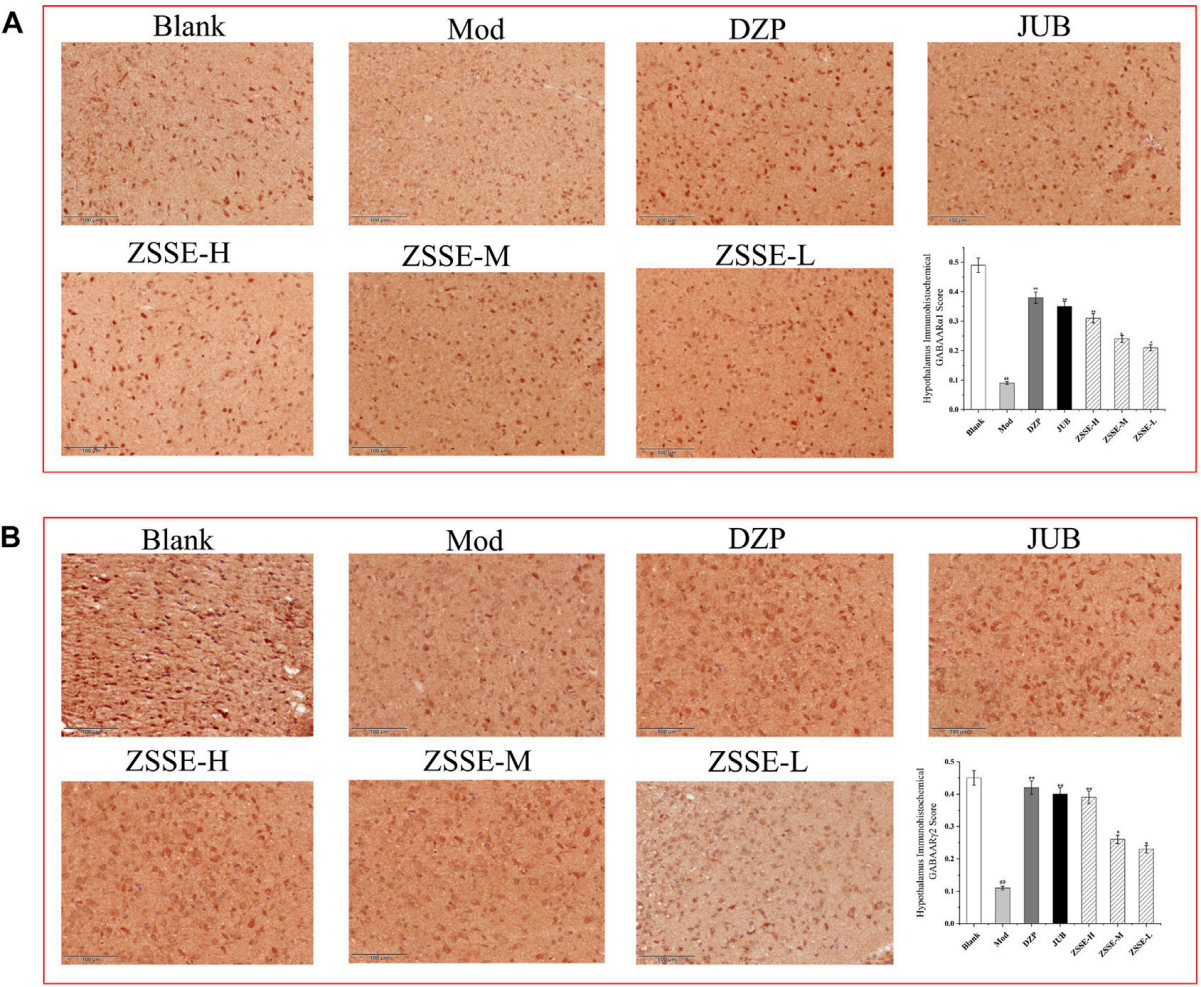
Effects of ZSSE on the expression of GABA_A_Rα1 and γ2 in the hypothalamus of PCPA-induced insomnia rats (n = 5). The expression of GABA_A_Rα1 receptor in the hypothalamus of PCPA-induced insomniac rats **(A)**; expression of GABA_A_Rγ2 receptor in the hypothalamus of PCPA-induced insomniac rats **(B)**. Blank: normal control, normal rats administered with 0.9% saline; Mod: PCPA-induced insomnia in rats, administered with 0.9% saline; DZP: PCPA-induced insomnia in rats, administered with 1.5 mg/kg/d diazepam; JUB: PCPA-induced insomnia in rats, administered with 20 mg/kg/d jujuboside B; ZSSE-H: PCPA-induced insomnia in rats, administered with 13.5 mg/kg/d ZSSE; ZSSE-M: PCPA-induced insomnia in rats, administered with 9.01 mg/kg/d ZSSE; ZSSE-L: PCPA-induced insomnia in rats, administered with 4.50 mg/kg/d ZSSE. Data are presented as mean ± S.D. (n = 5). Compared with the Blank group ^*^ *p* < 0.05, ^**^ *p* < 0.01; compared with the model group ^#^ *p* < 0.05, ^#^ *p* < 0.01.

As shown in Figure 9, under a light microscope, the cytoplasm of GABA_A_Rα1- and GABA_A_Rα1γ2-positive cells in the hypothalamus of the rats was stained brown-yellow. The normal control group exhibited more GABA_A_Rα1-and GABA_A_Rα1γ2-positive cells with a darker cytoplasm. In the model control group, the number of GABA_A_Rα1- and GABA_A_Rα1γ2-positive cells in the hypothalamus decreased notably, and the cytoplasm became lighter. One week after the intervention, the number of positive cells in the ZSSE administration group and the positive control group increased to different degrees, and the staining became darker. All the results showed that the average optical density of the positive cells in the model control group was significantly lower than that in the normal group. Compared with the model control group, the average optical density of the positive cells in each dose group was significantly higher.

### Effect of ZSSE on GABA_A_Rα1 and GABA_A_Rα1γ2 expression in the hippocampus of rats

**FIGURE 10:**
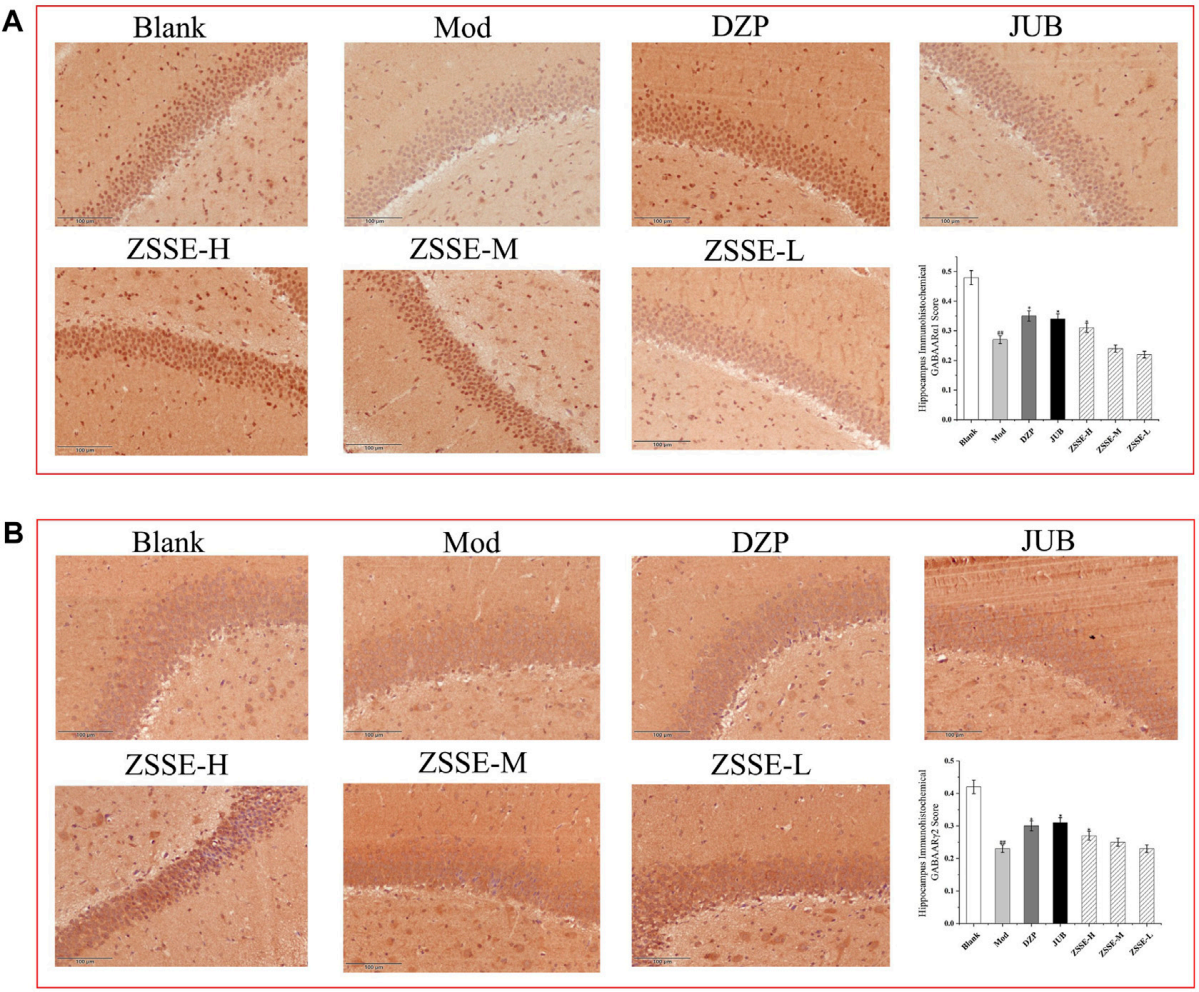
Effects of ZSSE on the expression of GABA_A_Rα1 and γ2 in the hippocampus of PCPA-induced insomniac rats (n = 5). The expression of GABA_A_Rα1 receptor in the hippocampus of PCPA-induced insomnia rats **(A)**; expression of GABA_A_Rγ2 receptor in the hippocampus of PCPA-induced insomnia rats **(B)**. Blank: normal control, normal rats administered with 0.9% saline; Mod: PCPA-induced insomnia in rats, administered with 0.9% saline; DZP: PCPA-induced insomnia in rats, administered with 1.5 mg/kg/d diazepam; JUB: PCPA-induced insomnia in rats, administered with 20 mg/kg/d jujuboside B; ZSSE-H: PCPA-induced insomnia in rats, administered with 13.5 mg/kg/d ZSSE; ZSSE-M: PCPA-induced insomnia in rats, administered with 9.01 mg/kg/d ZSSE; ZSSE-L: PCPA-induced insomnia in rats, administered with 4.50 mg/kg/d ZSSE. Data are presented as mean ± S.D. (n = 5). Compared with the Blank group ^*^ *p* < 0.05, ^**^ *p* < 0.01; compared with the model group ^#^ *p* < 0.05, ^#^ *p* < 0.01.

As shown in Figure 10, under a light microscope, the cytoplasm of GABA_A_Rα1- and GABA_A_Rα1γ2-positive cells in the rat hippocampus was stained brownish-yellow. The hippocampus of the normal control group exhibited more GABA_A_Rα1- and GABA_A_Rα1γ2-positive cells with a darker cytoplasm. In the model control group, the number of GABA_A_Rα1- and GABA_A_Rα1γ2-positive cells in the hippocampus decreased notably, and the cytoplasm became lighter. One week after the intervention, the number of positive cells in the ZSSE administration group and the DZP group increased to different degrees, and the staining became darker. All the results indicated that the average optical density of GABA_A_Rα1- and GABA_A_Rα1γ2-positive cells in the model control group was significantly lower than that in the normal group. Compared with the model control group, the mean optical density of GABA_A_Rα1-positive cells in the ZSSE 13.5 mg/kg group and the positive control group were increased, and the expression of GABA_A_Rγ2 in the ZSSE 13.5 mg/kg group was significantly different from that in the model group. No significant difference was found in the various dose groups of ZSSE.

## Discussion

The pathogenesis of insomnia is extremely complex. It has been shown that insomnia is closely related to the hypothalamus and the hippocampus, and extensive hypothalamic damage leads to complete insomnia (Yamagata et al., 2021; Bajaj and Kaur, 2022). Previous studies have also shown that when hippocampal cells are damaged and the volume of the hippocampus shrinks to a certain extent, the rapid eye movement (REM) sleep time can be reduced (Forouzanfar et al., 2021; Tapp et al., 2022). Based on our results, ZSSE can effectively improve and recover the damaged nerve cells in the hypothalamus and hippocampus of insomnious rats. Moreover, ZSSE has a good neuroprotective effect as shown by the HT22 nerve cell protection test *in vitro* and the classic pathological analysis of the hypothalamus and hippocampus of rats with insomnia induced by PCPA *in vivo*. On this basis, to further confirm the mechanism of ZSSE in improving sleep, we used ELISA, immunohistochemistry, and other molecular biological techniques.

Currently, studies on the mechanism of insomnia show that insomnia in humans is closely related to neurotransmitters and related nerve receptors in the brain. In a study of insomnia-related neurotransmitters, it has been found that the content of GABA, Glu, 5-HT, DA, and other neurotransmitters in the brain significantly affects the quality of human sleep (Chen et al., 2021; Jhawar et al., 2022). When the content of GABA in the brain decreases and the content of Glu increases, insomnia may occur (Li N. et al., 2018). When the content of 5-HT decreases significantly and the content of DA increases significantly, insomnia may also develop (Sun et al., 2020; Fagan et al., 2021). This study showed that the content of GABA and 5-HT in the hypothalamus and hippocampus of rats with insomnia decreased significantly, while the content of Glu and DA increased significantly. After administration, compared with the model group, the contents of GABA and 5-HT in the hypothalamus and hippocampus of insomnia rats in the ZSSE group increased significantly, while the contents of Glu and DA decreased significantly. These findings indicate that ZSSE can alleviate insomnia by regulating amino acids and monoamine neurotransmitters in the brain.

(Lorenz-Guertin et al., 2018) In studies of insomnia-related nerve receptors, it has been found that the GABA_A_ receptor is an important target for human insomnia. The current widely used anti-insomnia drugs, diazepam and benzodiazepines, were developed based on this receptor as a target (Bellelli et al., 2021; Ghit et al., 2021). Therefore, the present study focused on two important subunits of the GABA_A_ receptor, GABA_A_Rα1 and GABA_A_Rγ2. We found that ZSSE was able to significantly increase the expression of GABAα1- and GABAγ2 receptor-positive cells in the brain of rats with insomnia. It can be concluded that ZSSE can enhance the expression of GABA_A_Rα1 and GABA_A_Rγ2 and inhibit the excitation of neurons, thereby improving sleep.

In the comparative experimental study with diazepam, an anti-insomnia drug widely used in clinical practice, the content of GABA, Glu, DA, and 5-HT in the ZSSE dose group and the diazepam group had the same change trend, and there was no significant difference. It can be concluded that the mechanism of action of ZSSE and diazepam in treating insomnia may be similar, and they can both treat insomnia by regulating the content of inhibitory neurotransmitters and excitatory neurotransmitters. Further neuroreceptor studies found that the ZSSE dose group and the diazepam group showed a significant increase in the expression of GABA_A_Rα1- and GABA_A_Rγ2 receptor-positive cells in the brain of insomnia rats, which is consistent with the mechanism of action of diazepam on GABA_A_ receptor targets reported in the literature.

As a classic benzodiazepine drug, diazepam is widely used for the clinical treatment of insomnia, sedation, and the treatment of anxiety disorder. However, it is worth noting that benzodiazepines can lead to daytime dysfunction; affect an individual’s performance, cognition, and memory; and even increase the risk of accidents. The long-term use of benzodiazepines easily causes addiction, with tolerance and evident withdrawal symptoms. Therefore, it is not recommended to use benzodiazepines for the long-term treatment of insomnia but only for short-term emergency use. Through the observation of the general state of insomniac rats in this study, we found that the weight of these rats decreased significantly after modeling, and the weight of each group began to rise after 2 days of drug intervention, but the rate of weight increase in the ZSSE dose group was better than that in the diazepam group. After 5 days of administration, the rats in the diazepam group had daytime sleepiness; their amount of food and water intake was lower than that of the rats in the ZSSE dose group; and their response to external stimuli was slower than that of the rats in the ZSSE dose group. In contrast, the ZSSE dose group was significantly better than the diazepam group in terms of weight, hair color change, mental state, ability to respond to external stimuli, and daily activity. It can be concluded that ZSSE does not affect the daytime activity and reaction ability of animals while improving insomnia, so it may be a potential drug to treat insomnia.

Natural medicines or Chinese medicines have shown excellent therapeutic effects due to their multiple-target characteristics of multiple ingredients. The active ingredients of ZSSE were analyzed by combining HPLC and LC/MS. A total of 35 compounds were identified. To further clarify the material basis of ZSSE efficacy, classical receptor targets of benzodiazepines were selected for molecular docking. The docking results showed that compounds jujuboside A, jujuboside B, spinosin, hesperidin, quercetin, rutin, puerarin, and ferulic acid have strong binding ability with related pathways, thereby playing a role in improving sleep. Furthermore, jujuboside A and jujuboside B exhibited the most prominent binding ability, so they might be the main active substances from ZSSE for improving sleep. This is consistent with the previous results from the literature that jujuboside A and jujuboside B can effectively alleviate insomnia (Wang et al., 2012; Has and Chebib 2018). The aforementioned results showed that the pharmacological effect of ZSSE on improving sleep could be attributed to the comprehensive effect of various components of ZSSE, which reflects the multicomponent and multitarget characteristics of traditional Chinese medicine.

## Conclusion

In this study, HPLC and HPLC-MS were performed to identify the active ingredients of ZSSE. Moreover, molecular docking technology was used to prove that the effective substances of ZSSE for improving sleep are jujuboside B, jujuboside A, spinosin, and other chemical components. Combined with molecular biological techniques such as ELISA and immunohistochemistry, the mechanism and pharmacologic basis of ZSSE in improving sleep were discussed. ZSSE can improve sleep by activating the expression of GABA-specific receptors GABA_A_Rα1 and GABA_A_Rγ2 in the hypothalamic GABA system pathway. Furthermore, ZSSE exhibits the advantages of multiple ingredients, multiple targets, and multiple pathway treatment in comparison with diazepam. Specifically, ZSSE exhibits a unique prevention and treatment effect without notable adverse reactions or drug tolerance after the long-term use. Therefore, ZSSE is an active substance extracted from natural plants to improve sleep, which can be used as the raw material for the development of drugs to prevent and treat insomnia in the future.

## Data Availability

The original contributions presented in the study are included in the article/Supplementary Material; further inquiries can be directed to the corresponding authors.
